# Diel and tidal rhythms drive fish acoustic communities in a European kelp forest

**DOI:** 10.1186/s12862-026-02521-z

**Published:** 2026-05-02

**Authors:** Marine Ethève, Pierre Thiriet, Gaëlle Legras, Philippe Lenfant, François Bourrin, Lucia Di Iorio

**Affiliations:** 1 Université de Perpignan Via Domitia, Centre de Formation et de Recherche sur les Environnements Méditerranéens, CNRS, UMR 5110, 52 avenue Paul Alduy, Perpignan, 66860 France; 2 UAR PatriNat (OFB, MNHN, CNRS, IRD), Station Marine de Dinard - MNHN, 38 rue du Port Blanc, Dinard, 35 800 France

**Keywords:** Biophony, Passive acoustic monitoring, Marine habitat, Fish sounds, Environmental drivers, Soundscape, Underwater visual census, Temporal variability, Fish assemblages

## Abstract

Changes in marine animal communities shape ecological processes and ecosystem functioning. Monitoring temporal community dynamics is increasingly important under global change, yet remains challenging because community fluctuations can arise from multiple natural processes and are difficult to assess. Passive acoustic monitoring of signals produced by marine fauna offers a non-invasive means of tracking community dynamics, providing continuous, high-resolution data that capture temporal patterns often missed by traditional methods. Despite their potential as proxies for macrofaunal assemblages and associated dynamics, the responses of acoustic communities to environmental variability in marine ecosystems remain largely unexplored. This study aimed to characterize fish acoustic communities in a tidal European kelp forest and identify the environmental drivers shaping their temporal variability. Continuous acoustic recordings were combined with environmental measurements and underwater visual surveys to address these objectives. Generalized linear models revealed that diel and tidal cycles were the dominant drivers of acoustic activity, diversity, and community composition during the summer study period. Other environmental variables had weaker effects, reflecting the fauna’s adaptation to marked short-term fluctuations in this dynamic ecosystem. Acoustic activity and richness increased at low water height, contrasting with higher visually observed fish abundance at high water height. This discrepancy suggests that sound production reflects behavioural interactions rather than fish abundance alone. The greater number of sound types (26) relative to observed species (19) indicates either behavioural sound diversity within species or the presence of undocumented soniferous taxa. Calmer sea conditions also promoted higher vocal activity and acoustic richness. This study provides an unprecedented description of kelp forest acoustic communities in Europe, demonstrating the value of ecoacoustics to complement visual surveys for capturing natural variability and establishing essential baseline information for detecting long-term ecological shifts in these climate-sensitive habitats.

**Graphical Abstract:**

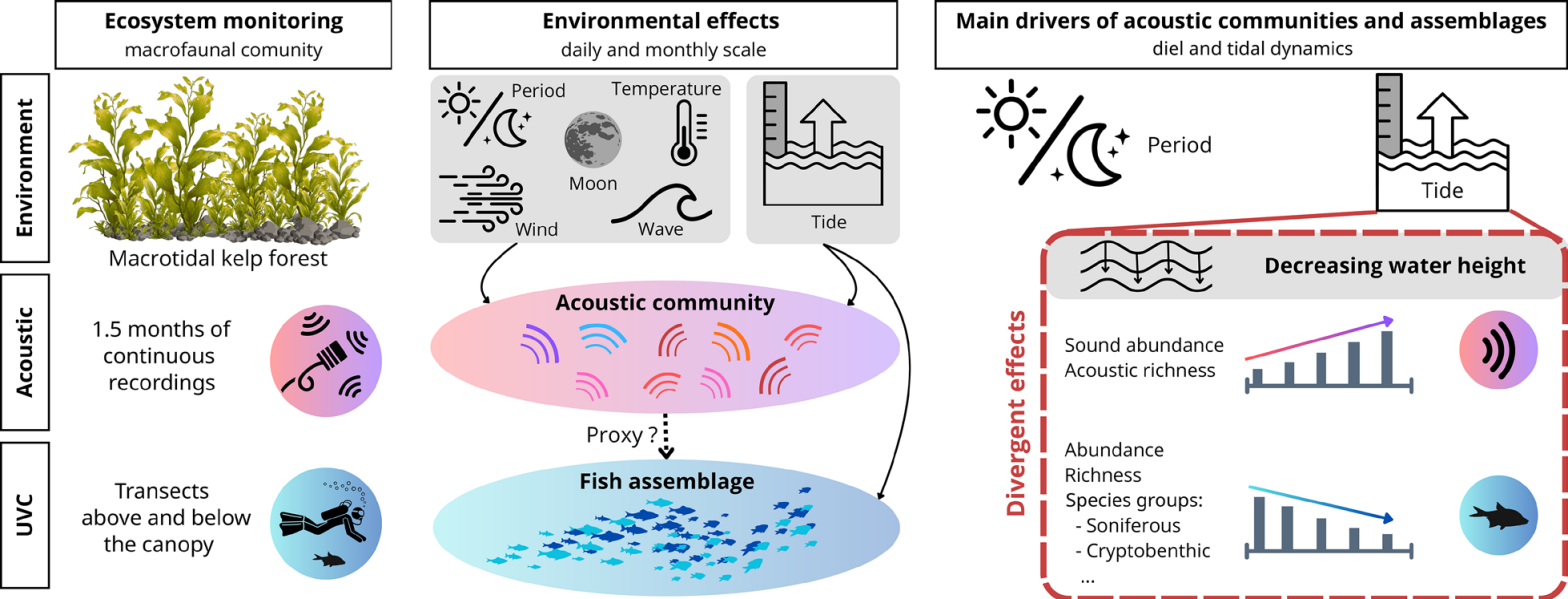

**Supplementary Information:**

The online version contains supplementary material available at 10.1186/s12862-026-02521-z.

## Introduction

Marine ecosystems host diverse biological communities that function as interconnected networks, where each species contributes to key ecological processes, through interactions such as competition, facilitation (e.g. habitat provision), or predation [1–3]. These marine communities play a critical role in the structure of ecosystems as they participate in key processes such as nutrient cycling, and primary and secondary productivity [4–6]. Changes in community structure may therefore impact ecosystem functioning. However, there is a lack of knowledge on the natural temporal variability in community dynamics shaped by environmental and/or chronobiological cycles such as diel, tidal, and lunar rhythms. Understanding this variability is essential in the context of global change. For example, the frequency and intensity of extreme events such as marine heat waves or storms, or alterations in acidification and ocean circulation patterns influence behaviours and interactions, with cascading effects on ecosystem functioning [7–13]. Documenting and monitoring community dynamics at temporal scales relevant to these processes is therefore critical. However, traditional biodiversity assessments are often discrete and conducted over short timeframes, limiting their capacity to capture temporal variability or to characterize the dynamics of community changes [14, 15]. Coarse temporal resolution may also overlook fine-scale fluctuations that can signal emerging shifts in community organisation [16, 17].

In recent years, there has been growing interest in the use of environmental sounds to investigate ecological and macrofaunal community complexity [18]. Animal sounds convey information on biodiversity, species presence, behaviours (e.g., feeding, courtship, resource defence), and interactions (communication within and between species) [19–21]. Recording these sounds is therefore an effective, non-invasive way of acquiring quantitative information on variations or changes in macrofaunal communities. Because acoustic acquisition is continuous and operates over long periods, it offers high temporal resolution and coverage, making it effective for detecting ecosystem dynamics and potential changes in assemblages induced by environmental factors [22, 23].

Fish and invertebrate sounds are dominant components of marine soundscapes [24–26]. However, only recently their diversity has been considered at the community level, rather than solely at the species level [27–30]. Fish produce a wide variety of sounds associated with behaviours such as courtship, spawning, and feeding activities [31–33]. Collectively, all these sounds form acoustic communities that have been shown to reflect different habitats [29, 34–36], their condition [37–39] and macrofaunal biodiversity [28, 40, 41]. As sounds reflect species presence and behaviours, acoustic communities hold strong potential as proxies to examine temporal changes in macrofaunal assemblages. However, despite their importance for understanding the effects of climate change on marine macrofaunal assemblages, the relationship between environmental variability and acoustic communities remains undocumented. Environmental factors can influence species composition and behaviour (e.g., calling activity or habitat use), which are directly reflected in the biological component of soundscapes [42, 43]. Understanding how acoustic diversity and activity respond to these factors is therefore essential for characterising natural ecoacoustic variability and evaluating potential effects of environmental fluctuations and extreme events on animal communities.

In this study we investigate how fish acoustic communities respond to temporal environmental variability within a tidal European kelp forest. Kelp forests are biodiversity hotspots that provide numerous ecosystem services and play an essential ecological role as nurseries, feeding areas, and by protecting fauna against physical stress [44, 45]. Yet, increasing temperatures affect community structure and associated macrofaunal assemblages [46, 47], which is why these ecosystems are considered highly vulnerable to global warming [48–50]. Similarly to terrestrial forests, a modified acoustic behaviour in a community in the face of environmental pressure can reflect changes in macrofaunal assemblages and habitat condition [51–54].

In shallow waters, tidal cycles further expose kelp forests and their associated fauna to strong fluctuations in water height, tidal currents and wave action [55, 56], creating pronounced, natural short-term variation in environmental variables (e.g., temperature). This makes kelp forests particularly valuable to study natural variability in macrofauna assemblages in relation to environmental factors and chronobiological cycles. However, the soundscapes of kelp forests are poorly documented worldwide (two in the USA [37, 57] and one in Australia [58]) and no studies have provided a description of macrofaunal acoustic diversity [59].

The objectives of this study are to provide the first description of acoustic communities in this important-yet-understudied habitat, to identify the main environmental drivers structuring their temporal variability, and to examine how acoustic patterns relate to fish assemblages. By combining passive acoustic monitoring and visual surveys, this study explores how environmental factors and chronobiological cycles drive animal communities in a dynamic coastal ecosystem.

## Materials and methods

### Data collection

#### Study site and acoustic recordings

**Fig. 1:**
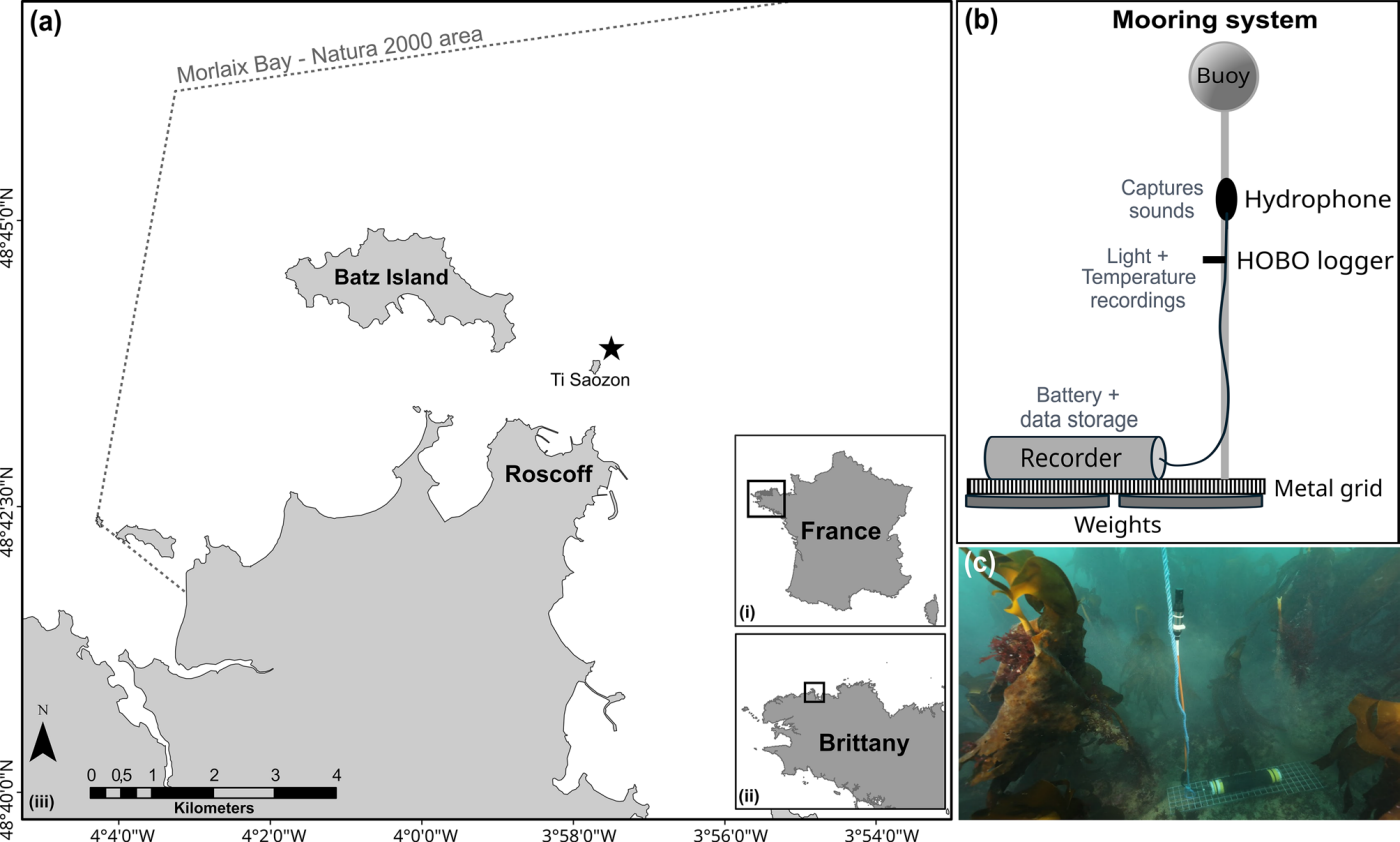
Location of the acoustic study site and acoustic mooring system. (**a**) Location of the study site near Roscoff, Brittany (France). Map generated in ArcGIS® 10.8. (**b**) Schematic representation of the mooring system. (**c**) Photo of the recorder deployed in the kelp forest. Photo credit: Wilfried Thomas - Roscoff Biological Station, 2023

Data was collected within the Traversing European Coastlines (TREC) expedition as part of the BIOCEAN5D project (HORIZON-CL6-2021-BIODIV-01-03). Acoustic recordings were obtained from a subtidal rocky reef ecosystem dominated by a kelp forest near Roscoff (48.73915° N −3.96735° E), Brittany, northern France (Fig. 1a). The study site was situated in a shallow, rocky region characterized by an extensive and dense kelp forest exposed to the effects of semi-diurnal tides within a macrotidal environment (tidal ranges > 8 meters).

This location falls within the Natura 2000 area of the Morlaix Bay. The kelp forest belt around Roscoff and Batz Island (Fig. 1a) is particularly noteworthy for its high biodiversity [60]. The shallow reefs and seabeds in this region support a rich array of flora and fauna that have been documented for over a century by the Roscoff Biological Station. The kelp forest is composed of various Laminaria species [61], including *Laminaria hyperborea* (dominant species) (Foslie, 1884), *Laminaria ochroleuca* (Bachelot de la Pylaie, 1824), *Laminaria digitata* (J.V. Lamour., 1813), and *Saccorhiza polyschides* (Batters, 1902).

The recording system consisted of a Colmar® GP 1516 hydrophone (High Tech Inc., receiver sensitivity: −170 dB re 1μPa/V, flat frequency response: 5 Hz − 35 kHz) connected to a SYLENCE-LP440 solid state recorder (RTSYS®, 128 kHz sampling rate, 24-bit rate). The mooring system consisted of a weighted grid on which the recorder was fixed (Fig. 1b–c). It was positioned within the forest at a depth of approximately 10 meters relative to chart datum (CD). The hydrophone was suspended one meter above the seafloor. Data were acquired with a continuous sampling regime between July 25^th^ and September 14^th^, 2023. Compared to snapshot (1 or 5 minutes) recordings commonly used in fish bioacoustics studies [62], continuous recordings are better suited for soundscape analysis and increase the probability of capturing acoustic diversity and its dynamics [20]. As biological sounds in kelp forests have been poorly characterized, we chose to study the summer period to capture the highest possible diversity. Studies indicate increased acoustic activity in summer, and some of the most abundant species in North Atlantic kelp forests are more prevalent during this season [57, 63]. This period is also less affected by strong winds, which are common in the region and can mask biological sounds.

A temperature (°C) and light (lux) logger (HOBO Pendant ® Temp/Light, 64K, UA-002-64, Onset Computer) was attached 20 cm below the hydrophone, logging once every 30 minutes during the entire sampling period.

#### Environmental data

To analyse the effect of environmental variables on fish acoustic communities, in addition to temperature and light, four quantitative variables, known to induce changes in macrofaunal assemblages [64–66], were selected and extracted from various data sources detailed in Table 1. These include wind speed and wind direction (calculated from the northward and eastward wind vector components using formulas (1) and (2)), significant wave height (used as a proxy for swell) and water height (correlated to tide).

**Table 1:** Summary of the six selected environmental variables and their respective sources. Data obtained from in situ HOBO sensor, Copernicus Marine Service [67, 68] and SHOM (Service Hydrographique et Océanographique de la Marine [69])

Variable	Significance	Product	Dataset	Nature	Spatial resolution	Temporal resolution
**Data from HOBO sensor (deployed on site: −3.96735 E 48.73915 N)**						
Temperature	Temperature at 10 m depth relative to CD (°C)			Measured in situ		Hourly
Light	Light at 10 m depth relative to CD (Lux)			Measured in situ		Hourly
**Data from Copernicus Marine Service - Data Store (MyOcean Viewer GPS point: −3.96735 E 48.73915 N)**						
Wind speed Wind dir.	Wind speed (m.s^−1^) and wind direction (°), measured from northward and eastward speed vector (m.s^−1^)	WIND_GLO_PHY_L4_MY_012_006	cmems_obs-wind_glo_phy_my_l4_0.125deg_PT1H	Numerical models + Satellite observations	0.125° × 0.125°	Hourly
Sign. wave height	Sea surface significant wave height (m)	NWSHELF_REANALYSIS_WAV_004_015	MetO-NWS-WAV-RAN	Numerical model + reanalysis	0.0135° × 0.0303°	Hourly
**Data from SHOM – Roscoff Station (−3.965679 E 48.718426 N)**						
Water height	Water height (m) based on hydrographic zero			Data validated		Hourly

Estimation of wind speed *V* (m.s^−1^)

1 $$V = \sqrt {\left( {v_n^2 + v_e^2} \right)} $$

Estimation of wind direction $\theta$ (degrees)

2 $$\theta = {\rm{atan}}2({v_e},{v_n}) \times \left( {{{180} \over \pi }} \right)$$

Where ${v_n}$ is the northly wind component (positive if the wind is northward, negative if it is southward), ${v_e}$ is the easterly wind component (positive if the wind is eastward, negative if it is westward) and ${{180} \over \pi }$ allow to convert angle from radiant to degrees.

#### Visual census of fish assemblages

**Fig. 2:**
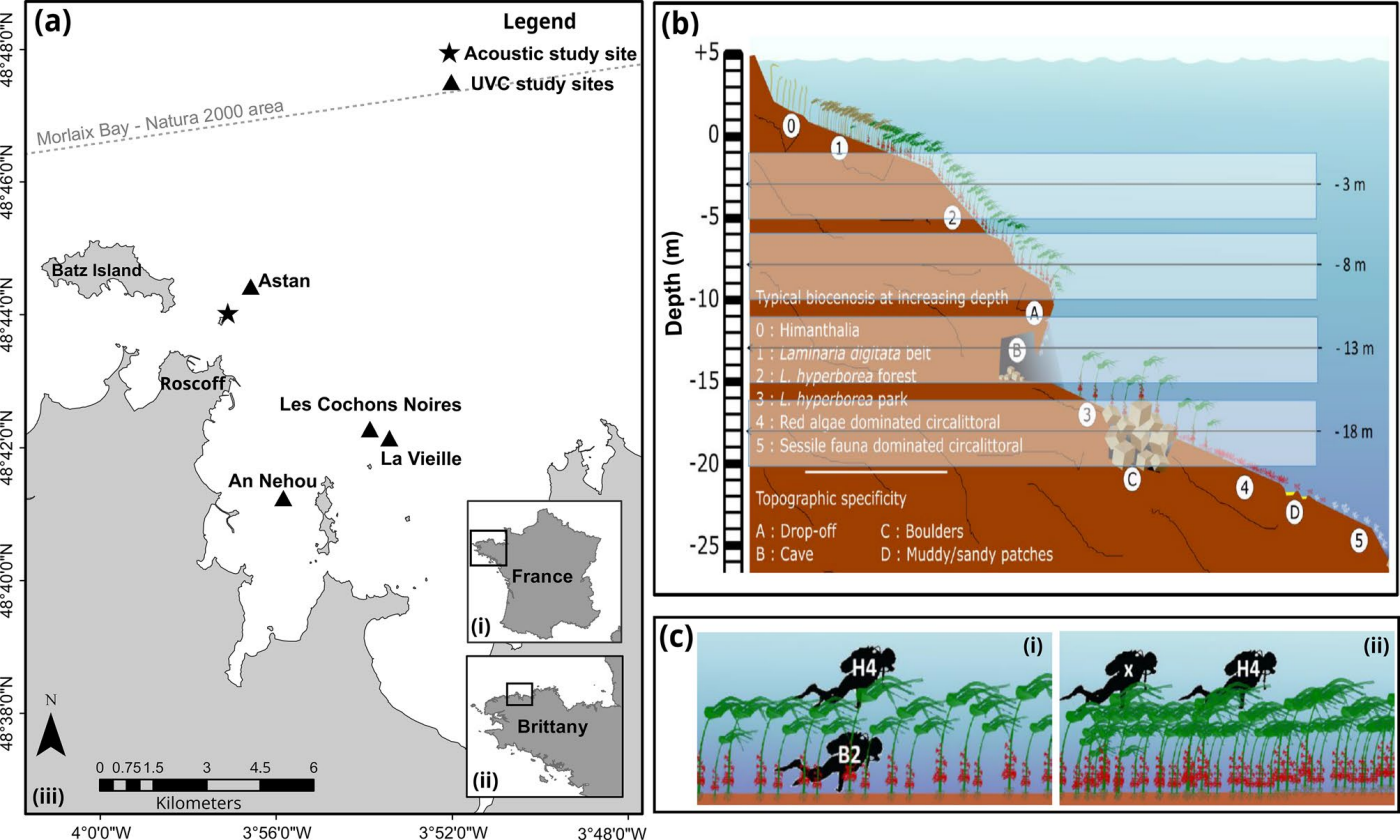
Location and sampling strategy for the UVC data. (**a**) Location of the four UVC sites near Roscoff, Brittany (France). Map generated in ArcGIS® 10.8. (**b**) Bathymetric strata with UVC surveys at each site. Each stratum spans a 4-meter bathymetric range (±2 m), centered around a target depth for transects (−3 m, −8 m, −13 m, and −18 m). (**c**) Illustration of diver roles and positions during transect surveys under (i) sparse kelp forests and (ii) in dense kelp forests. Figure (**b**) and (**c**) reproduced from the POCOROCH program – UMS PatriNat Report by Rey et al. [70]

Underwater Visual Census (UVC) data were obtained from the long-term monitoring network POCOROCH (POissons et Céphalopodes Côtiers des milieux ROcheux et des Herbiers de la façade Atlantique-Manche) at four kelp forest sites located around Roscoff: Astan (48.7455° N −3.96077° E), An Nehou (48.6932° N −3.94123° E), La Vieille (48.71047° N −3.90296° E), Les Cochons Noirs (48.71237° N −3.91076° E). This program is coordinated by PatriNat on the Atlantic-Channel facade. UVC sites were chosen for their proximity and ecological similarity to the acoustic monitoring site (Fig. 2a).

On the POCOROCH program, a standardized transect-based survey protocol has been implemented since 2016 (see protocol details in the report by Rey et al. [70]). Within each site, UVC transects were performed in kelp forests across bathymetric depths ranging from −1 m to −20 m CD (see depth strata centred around −3 m, −8 m, −13 m and −18 m CD in Fig. 2b) to encompass bathymetric variability of fish and habitats. For each sampling unit (i.e., transect), fish counts were conducted simultaneously by two scuba divers positioned vertically within the water column: one diver (H4), surveying above the kelp canopy, performed counts along a 30 × 4 m^2^ transect, while a second diver (B2), operating beneath the canopy, surveyed a 30 × 2 m^2^ transect. In conditions of high kelp density (Fig. 2c. ii), only the diver in H4 performed fish counts, whereas the second diver was limited to habitat characterization. For each transect, divers recorded species identity (or taxa at the finest possible level), fish body-size class, and individual abundance. Additional metadata included geolocation (latitude/longitude), date, depth, start and end time of the transect, and water height at the time of survey.

On the four UVC sites, the complete UVC dataset used for this study included a total of 61 transects (29 at the B2 position and 32 at the H4 position) conducted in 2018, 2021, and/or 2024, during the months of May, June, September, and/or October.

### Data analysis

#### Diversity of acoustic communities

Since most fish vocalize and hear in the low (<2000 Hz) frequency range [71, 72], audio recordings were down-sampled to 4 kHz. Because the sound diversity of kelp forests remains undescribed, acoustic recordings have been manually inspected to identify the different sound types. However, as analysing 1.5 months of data is too time-consuming, a two-step subsampling scheme was implemented. First, a subsampling was applied based on tidal coefficients. Tidal variation is known to influence both sound propagation and macrofaunal communities [73, 74]. Consequently, care was taken to balance the subsampling across tidal levels. Acoustic data were selected according to four tidal levels, defined using tidal coefficients and classified as follows: Low (~30–40), Medium (~50–60), High (~70–80) and Very High (>100). These levels were used to ensure the selection of representative 24-hour periods for analyses across varying tidal conditions. In total, 20 different 24-hour periods (from 12 a.m. to 12 a.m. to capture a complete night) were chosen as subsamples, corresponding to five periods per tidal coefficient level (Supplementary Fig. S1). Subsamples covered the whole study period and presented low-wind conditions to avoid acoustic masking of biological sounds [75].

The selected recording periods were visually and aurally analysed using Raven Pro 1.5 software (The Cornell Lab of Ornithology, Ithaca, USA). Spectrograms and associated waveforms were inspected to identify and manually select individual sounds (Fast Fourier Transform (FFT) = 512; overlap = 80%; Window type Hann). Given the time-intensive nature of manually annotating 24 hours of continuous recordings, a second subsampling scheme was implemented. Only the presence of specific sound types within a 10-minutes time bin was annotated. When multiple occurrences of the same sound type were detected, the instance with the highest signal‑to‑noise ratio, allowing clear identification, was selected. Sounds of low quality (very low signal-to-noise-ratio) or of ambiguous biological origin, accounting for less than 3% of the detected sounds, were excluded.

Observations of the recordings revealed a significant increase in flow-generated noise at the hydrophone during falling tides for days with High and Very High tidal coefficient levels, resulting in masking of fish sounds (Supplementary Fig. S2). To prevent sound detection bias during these periods with significantly reduced signal detectability, the corresponding hours were excluded from the acoustic dataset.

For the classification of the different sound types, a standardized, hierarchical method was used, adapted from Desidérà et al. [28]. Sound types were organized in 3 levels of complexity: 1) Single element, 2) Train (repetition of consecutive elements), 3) Sequence (repetition of trains (and elements)). The homogeneity of a sound was also considered: homogeneous sounds are composed of the same type of element or train, while heterogenous sounds are composed of different types of elements or trains. Fish sound identification was based on acoustic properties using five axes (adapted from Desidérà et al. [28]): (1) element type (e.g., frequency-modulated/pulse), (2) peak/centre frequency, (3) number of repetitions per call, (4) rhythm of repetition (no rhythm/constant/variable), (5) repetition speed. Existing sound type categories from the literature [28, 34, 76] were updated, and new categories were created to account for the observed diversity.

A total of 480 hours were recorded across the twenty 24-hour sampled periods. After excluding hours affected by flow-noise (*n* = 59), 421 hourly replicates were retained for analyses (Supplementary Table S1). For each replicate, the total acoustic relative abundance and richness were calculated.

#### Analysis of environmental parameters

The different analyses described in the following sections were performed in R studio (version 2025.05.0).

To analyse the impact of environmental variation on fish acoustic communities, it is essential to first assess how these variables changed during the study period (environmental dataset on Supplementary Table S2). All available hourly environmental data collected between 27/07/2023 and 14/09/2023 were used for the temporal series analyses to detect trends, cycles, and occurrence of extreme events. Time series were decomposed, using a multiplicative method (*stats* package, *decompose* function) to characterise the daily component. The method of Interquartile Range (IQR; detection of the value outside the 0.05 and 0.95% quantiles) was used to detect potential extreme events [77]. First, global trends and cycles over the 1.5-month study period were investigated using daily mean values. This daily aggregation reduces noise associated with hourly variability and facilitates the detection of longer-term (weekly) trends. Linear regressions with Mann-Kendall tests were used to assess these trends. The Autocorrelation Function (ACF) method was then applied on detrended series to identify broader periodic patterns occurring over several days or weeks. In a second step, a Cosinor model (*cosinor* package) using the original hourly data was applied to detect potential 24-hour and 12-hour cycles corresponding to diel rhythms and semi-diurnal tidal cycles respectively. This two-step approach allows to disentangle long-term temporal trends from shorter-scale periodic processes.

#### Influence of environmental variables on acoustic communities

##### Effect on acoustic relative abundance and richness

To analyse the impact of environmental variables, in addition to the six quantitative variables listed in Table 1, four qualitative variables were specified for each replicate: S24_num (number corresponding to the 24-hour period considered), Period (Day or Night), Tidal coefficient level (Low, Medium, High, Very High) and Moon phase (1^st^ quarter, Full moon, Last quarter or New moon). The Period was defined based on the civil sunrise and sunset times in Roscoff during the study period. Because sampling was organised in discrete 24-hour periods (from 12 a.m. to 12 a.m.), S24_num was used instead of calendar date. Both variables are equivalent categorical grouping factors accounting for between-day variability.

To assess the effect of environmental variables on acoustic relative abundance and richness, Generalized Linear Mixed Models (GLMMs; *glmmTMB* package) were applied. The protocol proposed by Zuur et al. [78] was used for building and validating mixed models. Preliminary analyses included checking for collinearity among explanatory quantitative variables using Variance Inflation Factors (VIF), assessing the distribution of response variables to guide the choice of appropriate error structures, and identifying potential zero inflation. The distribution of acoustic abundance and richness was assessed using a Kolmogorov-Smirnov test and with a QQ-plot. Because acoustic abundance and richness exhibited overdispersion, a negative binomial distribution was chosen. All the environmental variables were standardized due to differences in units. For the GLMMs analyses, the wind direction variable (a circular variable expressed in degrees) was decomposed into its circular components using sine and cosine transformations: wind_cos (north-south axis) = cos(θ) and wind_sin (east-west axis) = sin(θ), where θ represents the wind direction in radians (i.e., degrees × π/180). Exploratory analyses were also conducted to assess potential non-linear relationships between environmental variables and response variables. These analyses indicated that most relationships were generally linear or monotonic across the observed ranges, with only a weak non-linear pattern detected for light. However, this effect did not influence model fit. Consequently, a GLMM framework was retained as an appropriate and parsimonious modelling approach. Due to the nature of the dataset, temporal dependence (autocorrelation) was identified both between the 24-hour periods and among the hours within each period. To account for this temporal structure, we included a hierarchical random effect structure in the GLMMs [79], with S24_num (equivalent to dates) and Hours as nested grouping factors. This approach provides a flexible way to model implicit temporal dependencies and is robust to irregularities in the data, such as missing hours on certain days due to acoustic masking. This also allows the effects of environmental variables to be modelled independently of day-to-day and hourly variability. Model selection was based on stepwise comparison of nested models using the Akaike Information Criterion (AIC), and model validation was assessed through graphical residual diagnostics (*DHARMa* package), which allows evaluation of dispersion, zero-inflation, and overall model fit. Residual diagnostics confirmed that the models adequately captured the structure of the data.

##### Effect on acoustic community composition

To analyse the impact of environmental variables on the composition of acoustic communities, a Generalized Linear Latent Variable Model (GLLVM; *gllvm* package) was used. This approach allows for joint modelling of sound type responses to environmental variables, while accounting for unobserved ecological gradients or species interactions that may influence community composition. Each sound type was modelled with its own response to predictors, and latent variables served as random effects to capture structures not explained by the measured covariates [80]. Here we also included a random effect (S24_num) to consider temporal dependencies between days. Based on the distribution of each sound type, the Tweedie distribution was selected as it accommodates zero-inflated, continuous, and positively skewed data, which matched the characteristics of our acoustic abundance values. As for GLMMs, the circular component of wind direction (wind_cos and wind_sin) was used due to collinearity issues.

#### Tidal effects on fish assemblages

For each of the 61 UVC transects, the total fish abundance and the specific richness were calculated. The biomass was also calculated based on the allometric relation (Formula 3) that links fish length and their weight for each species:

3 $$W = a*{L^b}$$

Where *W* is the fish body weight (g), *L* the total length (cm), *a* and *b* are species-specific coefficients obtained from *FishBase* database [81].

Individuals not identified at the species level were not considered for the analyses. For each species, several characteristics were included in the dataset: the family, if the species is known to produce sounds, the mean adult length class, the trophic diet, the level of mobility, the cryptobenthic or nektobenthic nature of the species, and the observed species-specific length class (small (1/6–1/3), medium (1/3–2/3), and large (>2/3)), in relation to the maximum known length of each species (based on *FishBase* values [81]) (UVC dataset available on Supplementary Table S3). Fish individuals smaller than 1/6 of the species’ maximum length, often highly abundant, were discarded from the dataset since these early juvenile stages usually exhibit significant fine-scale spatio-temporal variability that typically introduces noise and reduces the statistical power of community-level analyses [82].

Since UVC data are discrete, water height and tidal coefficient levels (Low, Medium, High, c.f., Section “Diversity of acoustic communities”) were the only environmental variables that could be tested on fish assemblages on the B2 (under kelp canopy) and H4 (above kelp canopy) positions. Analyses were conducted on B2 (2 × 30 m^2^) and H4 (4 × 30 m^2^) separately due to the different sampling surface area.

Linear regressions were first performed to assess the effect of water height on fish abundance, richness, and biomass. Mann-Whitney rank comparisons were conducted on fish abundance, richness and biomass and also on the different fish characteristics previously mentioned. To do so, the continuous water height variable was categorized into three classes: low (<4.5 m), medium (4.5–6.5 m), and high (>6.5 m). Then, to assess the influence of water height and tidal coefficient on the composition of fish assemblages, a non-metric Multidimensional Scaling (nMDS) using Bray–Curtis dissimilarity (suitable for species abundance data and insensitive to joint absences) was performed on square-root-transformed abundance data (species × length class matrix). Environmental vectors were fitted into the ordination space (*vegan* package, *envfit* function).

## Results

### Acoustic diversity

Considering the presence/absence of sound types every 10 minutes across the 20 selected 24-hour periods, a total of 6172 sounds were annotated. The number of sound occurrences per hour varied between 2 and 40, with a mean value of 14.7 occurrences per hour (±6.79 sd (standard deviation), *n* = 421 hours). Hourly sound type richness varied between 1 to 17 (mean = 8 ± 3 sd, *n* = 421 hours).

**Fig. 3:**
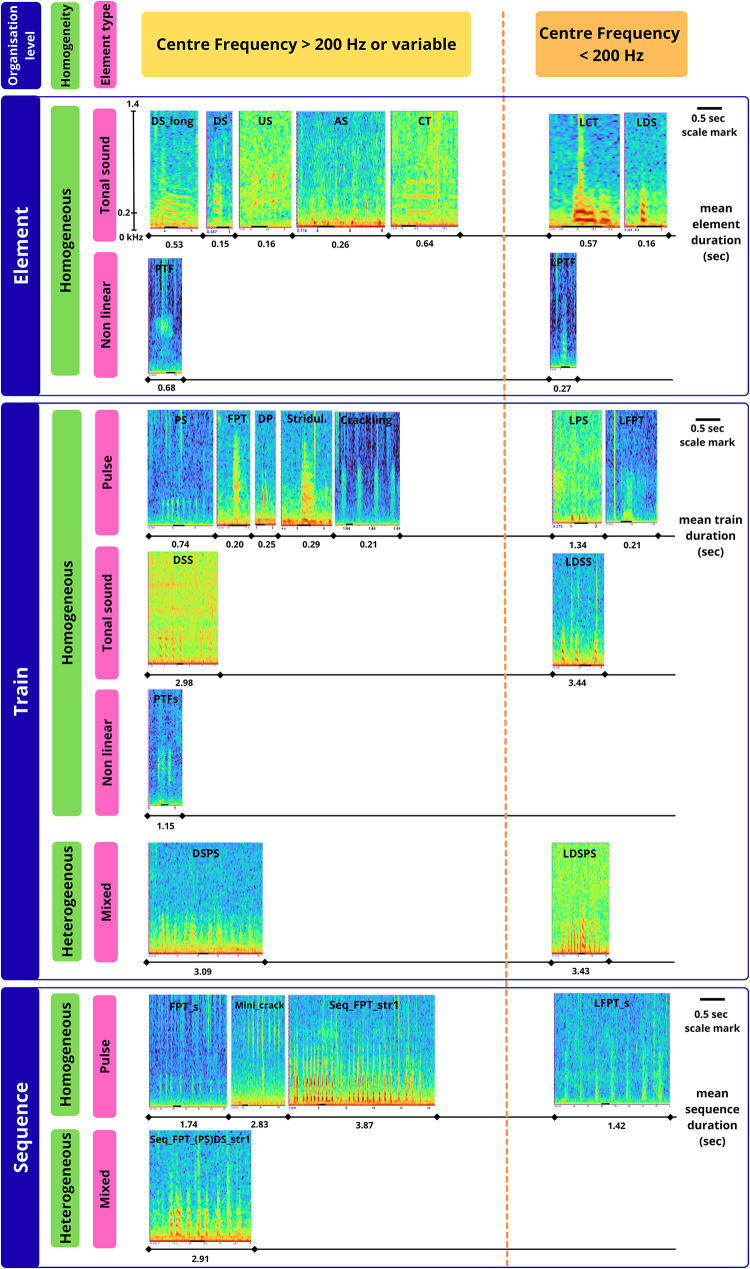
Spectrogram representation of the 26 different sound types. cf. Supplementary Table S4 for sound type abbreviations. Inspired by the representation of Puebla-Aparicio et al. [83]

A total of 26 different sound types were identified and are represented in Fig. 3 (detailed sound type descriptions in Supplementary Table S4). All the sound types were emitted sporadically, with some being more abundant than others, but none occurred in long series or as part of choruses. Most of these sounds are likely produced by fish, although some (approximately 4–5) could originate from invertebrates (i.e. Stridulation, Crakling), highlighting the current limitations in sound source identification. Only three of the 26 sound types displayed a highly stereotyped structure. The Stereotyped Sequence of Fast Pulse Trains 1 (Seq_FPT_str1) is a sequence of trains usually composed of 2 pulses (sometimes 3 or 4) repeating with a specific rhythm. The Seq_FPT_(PS)DS_str1 sound type is similar to the Seq_FPT_str1 but includes one or several downsweeps (DS). The Long Downsweep (DS_long) sound type is a frequency modulated sound around 200 Hz with a particularly long duration compared to the other sounds classified in the Downsweep (DS) sound type category.

**Fig. 4:**
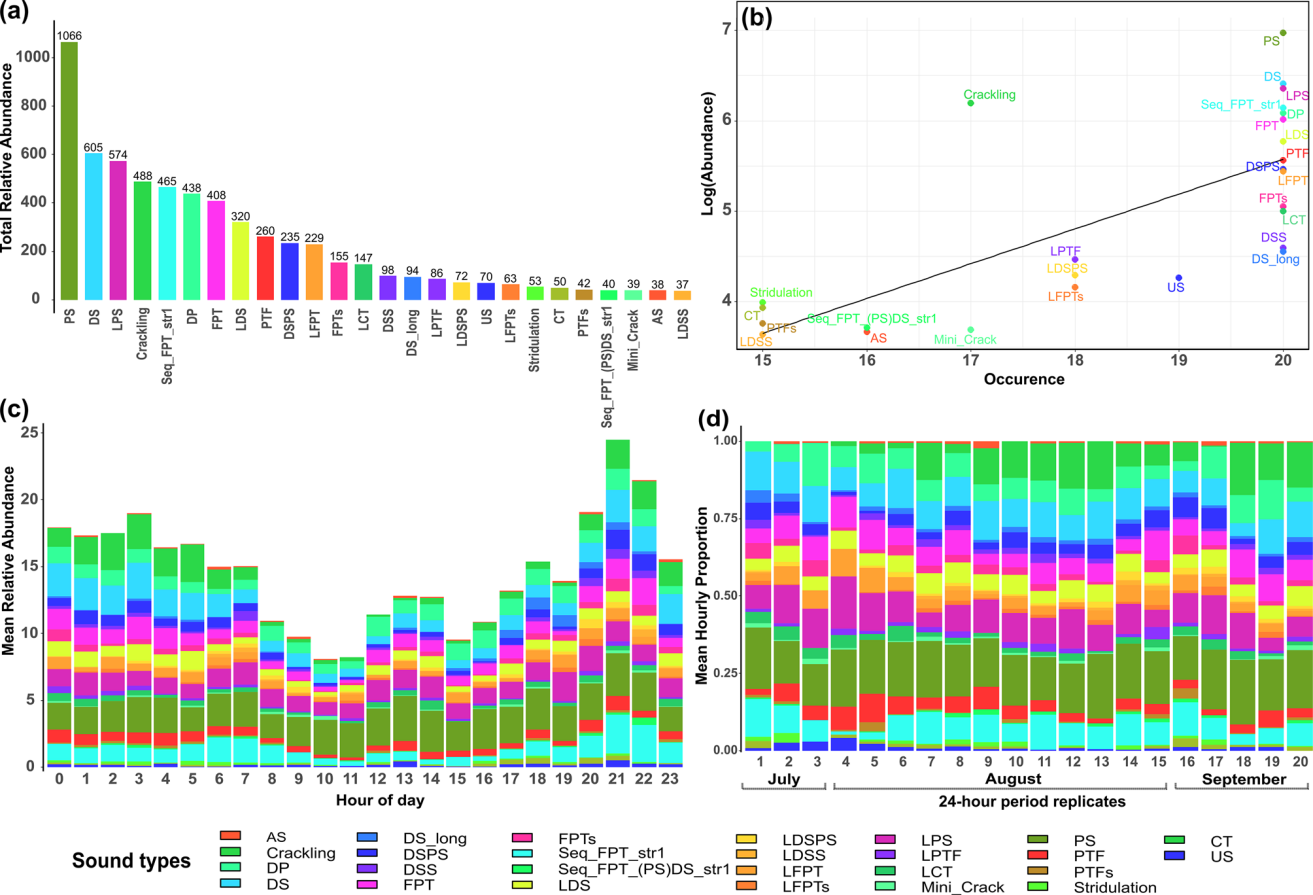
Macrofaunal acoustic communities of the kelp forest during summer 2023 (cf. Supplementary Table S4 for sound type abbreviations). (**a**) Distribution of the total acoustic relative abundance of each sound type from all the *n* = 421 hours. (**b**) Occupancy-abundance plot showing the abundance of sound types across all 24-hour periods sampled. Sound types on the top right were the most common, while those on the bottom left occurred only on some days. (**c**) Bar chart showing the acoustic diversity and relative abundance during hours of the day based on the identified sound types (indicated by distinct colours) and their mean relative abundances per hour. (**d**) Bar chart showing the acoustic diversity of each 24-hour period sampled based on the identified sound types (indicated by distinct colours) and the proportion of mean hourly relative abundances

Acoustic communities varied in the relative abundances of different sound types. The Pulse Serie (PS) sound type was the most abundant one (1066 annotations), while the least abundant sound type was the Low Downsweep Serie (LDSS) (37 annotations) (Fig. 4a). The three most abundant sound types, including the PS, the DS and the Low Pulse Serie (LPS), all with relative abundance over 500, were present on all sampled days. Other, less abundant sound types - i.e. the Downsweep + Pulse sequence (DSPS), the Low Fast Pulse Train (LFPT), the DS_long or the Downsweep Series (DSS) - were also present on all sampled days. Thirteen sound types out of the 26, showed relative abundances of less than 100 annotations overall, but all the sound types were detected in at least 15 out of the twenty 24-hour periods (Fig. 4b). A diel pattern was observed with an increase in acoustic relative abundance and richness during the night (Fig. 4c). Over the study period, acoustic community composition was relatively stable and did not show marked variations as indicated by the colour bars in Fig. 4d.

### Variation of environmental variables

**Fig. 5:**
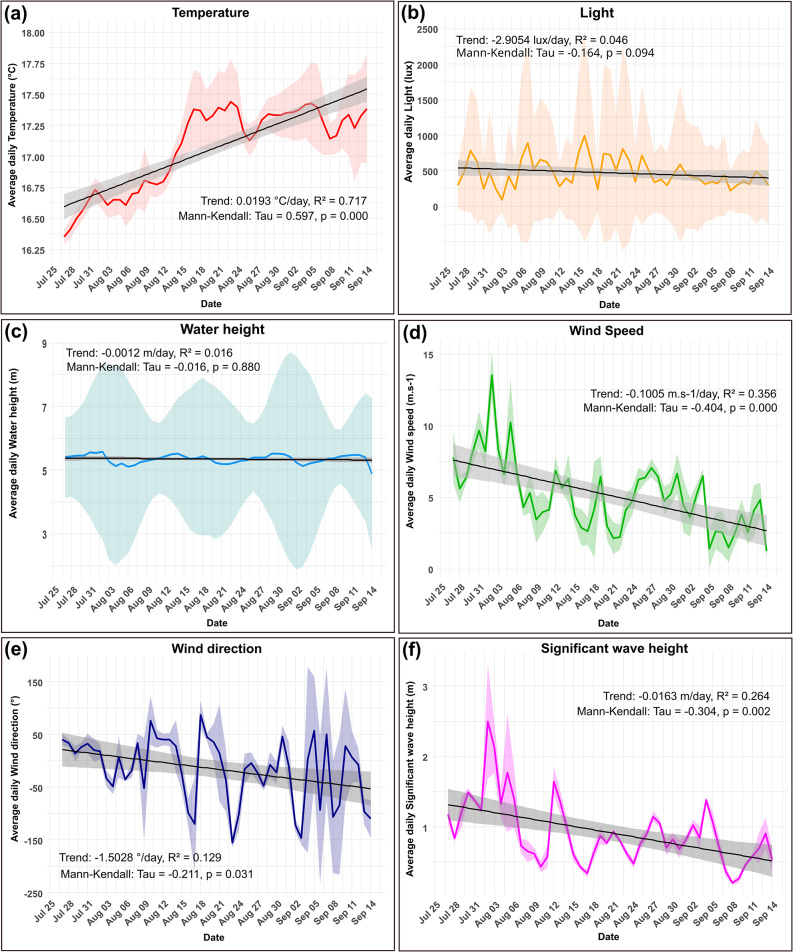
Temporal evolution of environmental variables (daily average ± sd) with linear trend analysis. Each panel shows the daily dynamics of an environmental variable measured between July 27^th^ and September 14^th^, 2023. A linear regression model (black line) with 95% confidence interval (grey band) is fitted to highlight overall trends. Results of the Mann-Kendall tests are indicated. (**a**) Temperature (°C; red). (**b**) Light (lux; orange). (**c**) Water height (m; blue). (**d**) Wind speed (m.s^−1^; green). (**e**) Wind direction (°; dark blue). (**f**) Significant wave height (m; magenta)

The temporal series of all six quantitative environmental variables were analysed to detect extreme events, trends and cycles. No extreme events were detected in any of the environmental variables studied. Significant temporal trends were found in four variables (Fig. 5): temperature increased from 16.3 °C to 17.9 °C by the end of the study period, wind speed globally decreased (from 5 to 12 m.s^−1^ to 0–5 m.s^−1^) and a shift in wind direction from N to NE was observed on average by the end of August. Finally, significant wave height decreased from 2 to 3 meters to less than 1 meter by the end of the study period.

Cycle analyses revealed a diel cycle in four parameters. For temperature and light, cycles were correlated with the day/night cycle. Although less significant, a diel cycle was also detected in wind parameters. For water height, two cycles were detected, a semi-diurnal cycle, corresponding to the two tides per day, and a monthly cycle linked to the variation of tidal coefficients.

### Effect of the environmental variable on acoustic richness and relative abundance

Results of the GLMMs with hierarchical random effects (hours within 24-hour periods) to test the effect of the different environmental variables on acoustic relative abundance and richness, are presented in Table 2. For both models, the random temporal effects explained a very small portion of the residual variance, suggesting limited inter- and intra-day unexplained variability.

**Table 2:** GLMMs results summary on the effect of environmental variables on acoustic relative abundance and richness. Models include a hierarchical random effect (Hours: S24_num) (*n* = 421 replicates). Significant *p*-value (<0.05) are written in bold. std. dev. = standard deviation

GLMMs best model results			Random effects		
Variables	z-value	p-value	Groups	Variance	Std. dev.
**Parameter: Acoustic relative abundance (Negative binomial distribution)**					
Tidal coeff. level - Medium	1.45	0.15	Hours : S24_num	1.096e^−8^	0.0001
Tidal coeff. level - High	−2.45	**0.01**	S24_num	5.691e^−3^	0.075
Tidal coeff. level - Very High	0.017	0.87			
Period - Night	5.09	**3.66e**^**−7**^			
Sign. wave height	−3.74	**0.0002**			
Light	−4.31	**1.66e**^-**5**^			
Water height	−5.23	**1.68e**^-**7**^			
Wind_cos	1.86	0.06			
Tidal coeff. level - Medium : Period - Night	−4.03	**5.52e**^**−5**^			
Tidal coeff. level - High : Period - Night	−0.39	0.7			
Tidal coeff. level - Very High : Period - Night	−3.19	**0.001**			
**Parameter: Acoustic richness (Negative binomial distribution)**					
Tidal coeff. level - Medium	1.30	0.19	Hours : S24_num	7.497e^−9^	8.659e^−5^
Tidal coeff. level - High	−1.43	0.15	S24_num	6.6046e^−3^	0.081
Tidal coeff. level - Very High	0.82	0.41			
Period - Night	4.30	**1.70e**^**−5**^			
Light	−4.29	**1.82e**^**−5**^			
Sign. wave height	−2.31	**0.02**			
Water height	−4.15	**3.37e**^**−5**^			
Wind_cos	1.59	0.11			
Tidal coeff. level - Medium : Period - Night	−3.60	**0.0003**			
Tidal coeff. level - High : Period - Night	−0.36	0.72			
Tidal coeff. level - Very High : Period - Night	−2.78	**0.005**			

Relative acoustic abundance was significantly influenced by several environmental variables (Table 2): Acoustic abundance significantly decreased with higher tidal coefficients (*p* = 0.01 for high tidal coefficient levels), particularly at night (significant negative interaction between Medium and Very high tidal coefficient and night period). In addition, higher water height (*p* < 0.0001), increased light levels (*p* < 0.0001), and greater significant wave height (*p* < 0.001) were all associated with a decrease in sound abundance. In contrast, acoustic abundance was significantly higher at night (*p* < 0.0001).

Acoustic richness followed a similar pattern. The negative effect of higher tidal coefficient was significant during the night (negative interaction between Medium and Very high tidal coefficient and night period). Furthermore, acoustic richness decreased with increasing water height (*p* < 0.0001), light levels (*p* < 0.0001) and significant wave height (*p* = 0.02). Acoustic richness was also significantly higher at night (*p* < 0.0001).

Overall, the most influential factors shaping acoustic relative abundance and richness were those related to diel cycles (night period and light levels) and tidal dynamics (tidal coefficient and water height) (Supplementary Fig. S3), highlighting the strong temporal structuring of acoustic communities in response to chronobiological rhythms.

### Effect of environmental variables on the composition of acoustic communities

**Fig. 6:**
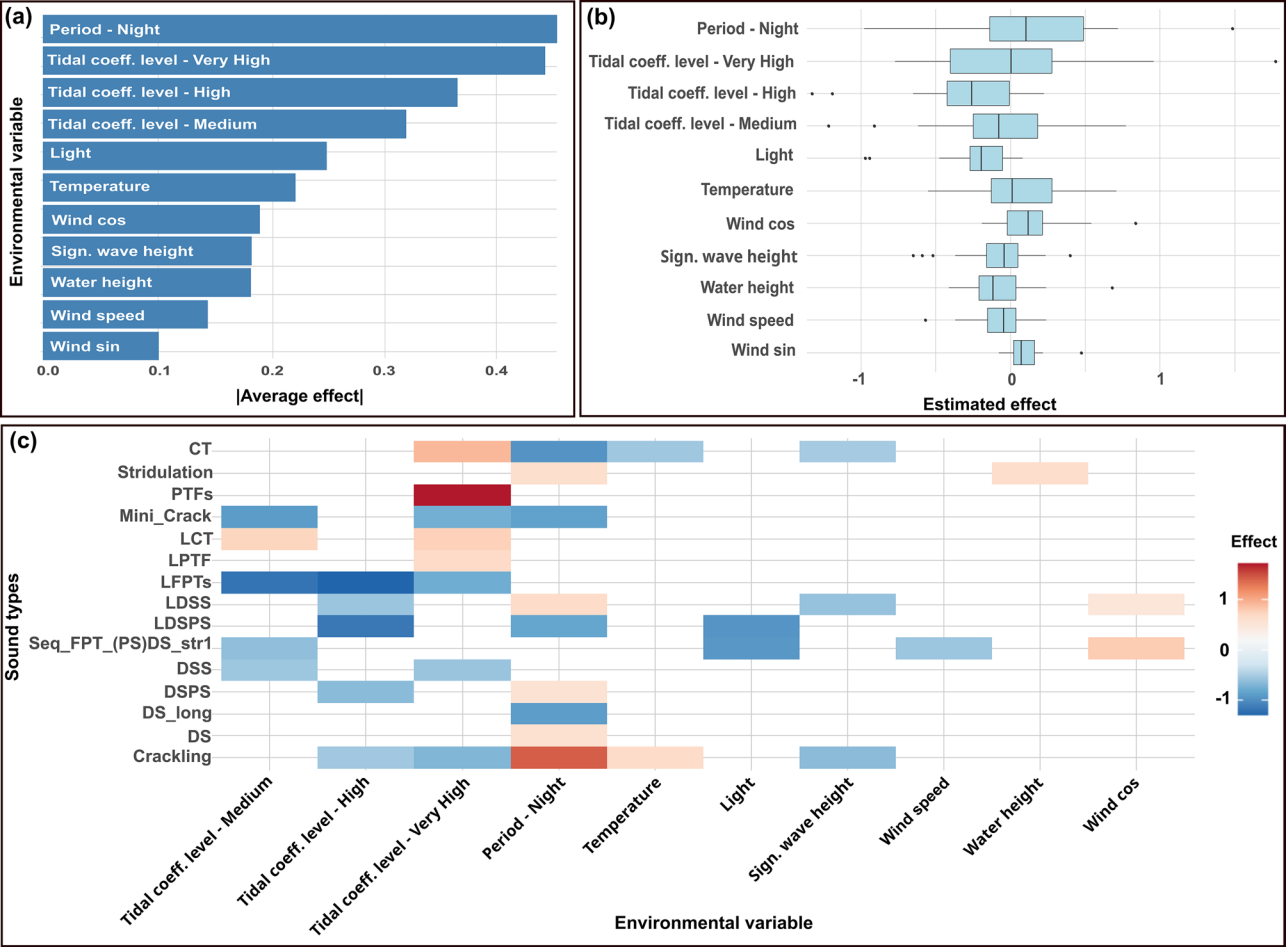
Global and individual effects of environmental variables on acoustic communities, resulting from the GLLVM analysis. (**a**) Barplot of the absolute average effect per environmental variable. (**b**) Boxplot of the distribution of effects per environmental variable. (**c**) Heatmap of the strongest individual effects (>|0.5|) of environmental variables on the different sound types. (cf. Supplementary Table S4 for sound type abbreviations)

The GLLVM used to test the effect of environmental factors on the composition of acoustic communities revealed that several environmental variables significantly contributed to the structuring of macrofaunal acoustic communities. Tidal coefficient (especially High and Very High levels) and diel period (Day/Night) showed the strongest average effects across sound types. Other variables, such as light intensity, temperature, and wind components had more moderate average effects (Fig. 6a). Whether variables had a positive or a negative effect depended on the sound type. However, some general trends emerged, such as an overall positive impact of night and the north and east winds, and a rather negative effect of hight tidal coefficients, wind speed, water height, and significant wave height (Fig. 6b). The heatmap of strong effects (|effect| >0.5; Fig. 6c) revealed that fifteen sound types showed strong associations with at least one environmental variable. Some sounds, such as the Time Frequency Bin series (PTFs), the LFPTs, the Crackling, the Constant Tonal (CT) or LDSPS, were particularly responsive to variables like tidal coefficient and diel period, while other sounds appeared to have little or no response to the covariates. Some sound types were influenced by several environmental variables such as the CT, the Crackling, the LDSS or the Seq_FPT_(PS)DS_str1 sound types. Instead, others were correlated with only one, such as the PTFs, the LPTF, the DS_long or the DS sound types. Moreover, two acoustically similar and close sounds, i.e., the Seq_FPT_str1 and the Seq_FPT_(PS)DS_str1 showed different responses. The Seq_FPT_(PS)DS_str1 sound type was correlated with tidal coefficient levels, light and wind parameters, while the Seq_FPT_str1 was not influenced by any variable.

### Effect of water height and tidal coefficient on fish assemblages

The fish assemblages on the four sites were composed of a total of 19 species. Their characteristics are detailed in Table 3. Among them, there were a majority of Labridae and Gobiidae, most individuals were of small sizes (adult size < 15 cm), invertebrate predators, and resident species with little mobility. Six of the 19 species have been reported to emit sounds (according to *FishSound* [84] and *Macauley Library* [85]).

**Table 3:** Summary of the 19 fish species and associated characteristics identified during the UVC transects. Four sampling sites were considered (Astan, an Nehou, La Vieille, Les Cochons Noirs). Maximum length value (cm) obtained from *FishBase* [81]. Pisc. = Piscivorous; Invert. = Invertebrate; Opport./Omni. = Opportunist/Omnivore

Species	Family	Sound producer	Adult length (cm)	Trophic diet	Mobility	Crypto-/nektobenthic	Max. length (cm)
*Pollachius pollachius*	Gadidae	Yes	>30	Pisc. predator	Wide-ranging	Nekto.	130
*Pomatoschistus flavescens*	Gobiidae	Yes	<15	Invert. predator	Resident	Crypto.	6
*Labrus bergylta*	Labridae	Unknown	>30	Invert. predator	Resident	Nekto.	65.9
*Symphodus melops*	Labridae	Yes	15–30	Invert. predator	Resident	Nekto.	28
*Thorogobius ephippiatus*	Gobiidae	Unknown	<15	Invert. predator	Resident	Crypto.	13
*Callionymus lyra*	Other	Unknown	<15	Invert. predator	Coastal mobile	Crypto.	30.5
*Centrolabrus exoletus*	Labridae	Unknown	15–30	Invert. predator	Resident	Crypto.	18
*Ctenolabrus rupestris*	Labridae	Unknown	<15	Invert. predator	Resident	Crypto.	18
*Gobius paganellus*	Gobiidae	Yes	<15	Invert. predator	Resident	Crypto.	13
*Labrus mixtus*	Labridae	Yes	>30	Invert. predator	Resident	Nekto.	40
*Mullus surmuletus*	Other	Unknown	15–30	Invert. predator	Coastal mobile	Nekto.	40
*Parablennius gattorugine*	Blenniidae	Unknown	<15	Opport./Omni.	Resident	Crypto.	30
*Zeugopterus punctatus*	Other	Yes	15–30	Invert. predator	Resident	Nekto.	25
*Parablennius ruber*	Blenniidae	Unknown	<15	Opport./Omni.	Resident	Crypto.	14.1
*Dicentrarchus labrax*	Other	Unknown	>30	Pisc. predator	Wide-ranging	Nekto.	103
*Trisopterus luscus*	Gadidae	Unknown	>30	Invert. predator	Coastal mobile	Nekto.	46
*Lepadogaster candolii*	Other	Unknown	<15	Invert. predator	Resident	Crypto.	7.5
*Tripterygion delaisi*	Other	Unknown	<15	Invert. predator	Resident	Crypto.	8.9
*Spondyliosoma cantharus*	Other	Unknown	15–30	Opport./Omni.	Coastal mobile	Nekto.	60

Fish in H4 (above the canopy) were not affected by water height nor the tidal coefficient levels, because linear regressions, NMDS, and Mann-Whitney comparisons did not reveal any significant effect.

**Fig. 7:**
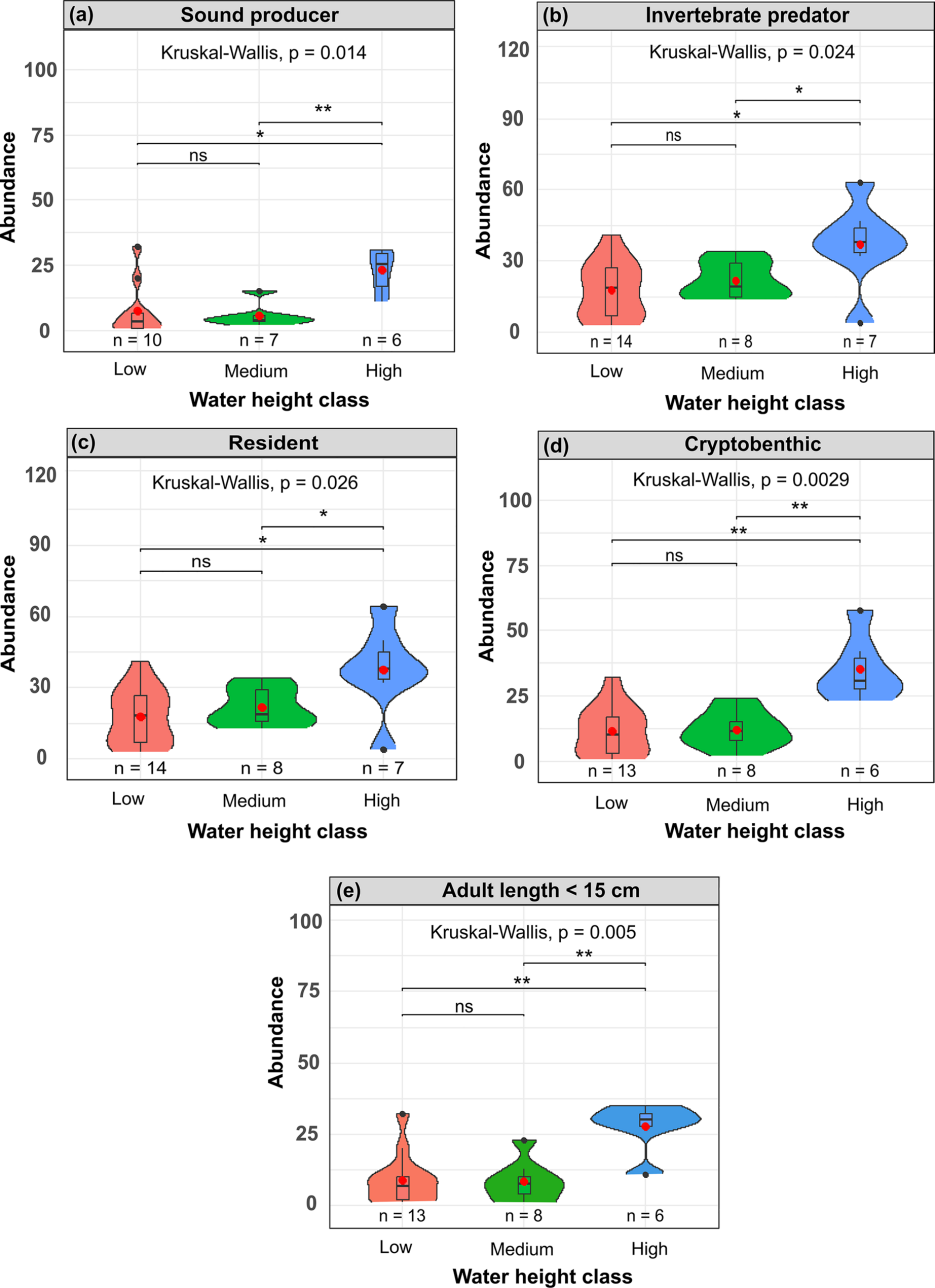
Variation in below-canopy fish abundance across water height classes for different species characteristics. (**a–e**) Violin plots showing the distribution of fish abundance by water height class (low: <4.5 m, Medium: 4.5–6.5 m, high: >6.5 m) for: (**a**) sound-producing species, (**b**) invertebrate predators, (**c**) resident species, (**d**) cryptobenthic species, and (**e**) adult length < 15 cm. Results of Kruskal–Wallis tests and Mann-Whitney pairwise comparisons are indicated (ns = not significant, * = *p* < 0.05, ** = *p* < 0.01). Number of transects in B2 position are indicated at the bottom for each crossed factor

However, for fish in B2 (below the canopy), linear regressions showed a significant positive effect of water height on fish abundance (*p* = 0.00152; adj. R^2^ = 0.29) and species richness (*p* = 0.0164; adj. R^2^ = 0.17), but not on total biomass (*p* = 0.421). NMDS analyses and subsequent fitting of environmental variables revealed a significant effect of water height on community structure (*p* = 0.012; r^2^ = 0.29). Moreover, Mann-Whitney analyses, exploring the responses of species according to their characteristics (c.f. Table 3), have highlighted significant effects of water height and to a lesser extent of tidal coefficient level on the distribution of certain categories of fish (Fig. 7). In fact, an increase in sound-producing species, invertebrate predators, resident, cryptobenthic and small species (adult length < 15 cm) was observed with increasing water height, and in particular for the “high” water height classes (>6.5 m) (Fig. 7).

## Discussion

This study offers an unprecedented, detailed description of macrofaunal acoustic communities within a kelp forest and provides a comprehensive assessment of how short-term environmental variability shapes their temporal dynamics. Diel and tidal cycles emerged as the primary drivers of acoustic activity, diversity and community composition during the summer study period. This suggests a strong natural cyclicity that governs the behaviour and possibly interactions of coastal fauna during summer. Tidal fluctuations, in particular, strongly influenced acoustic patterns, with sound production increasing as water height decreased. In contrast, the UVC data revealed an opposite trend with higher fish abundance and richness at high water height suggesting that greater species abundance does not necessarily translate into increased sound production.

Increased sound production at low water height may instead derive from enhanced behavioural interactions associated with fauna being concentrated into a reduced habitat volume [86–88], potentially promoting territoriality, courtship, or other social behaviours. Moreover, the fact that higher acoustic abundance and richness was observed during periods of low tidal coefficients, could also depend on the effect of current speed on fish behaviour. Significant wave height was negatively associated with both acoustic relative abundance and richness, suggesting that vocal activity tends to increase during calmer sea conditions [74], less exposed to strong recurrent tidal currents. This may induce the macrofauna to be more active, which could also result in increased acoustic activity. The role of tidal currents is likely more complex than captured by water height or tidal coefficient alone. Although the effect of current speed was not directly assessed, exploratory analyses (Supplementary Fig. S4) revealed variation in acoustic relative abundance and richness across tidal phases even at similar water heights. These observations suggest that current speed or current direction may modulate vocal activity, likely through effects on species behaviour [89]. Future studies should include direct measurements of current velocity during acoustic sampling to more accurately evaluate the influence of hydrodynamic conditions on sound production as well as species presence and abundance in shallow coastal tidal ecosystems.

Alternatively, the effect of tides on acoustic behaviour may be mediated by physical characteristics of the water column. Sound propagation likely played a minor role here as falling tides reduce both the propagation channel as well as the detection range of sounds. However, tidal-driven water movement affects sound propagation conditions due to significant variations in temperature and salinity [90], which in turn may alter the acoustic detectability of species vocalizations. Tidal currents may also introduce noise interference linked to increasing/decreasing water flow and turbulence [91] at the hydrophone, potentially masking biological sounds. However, acoustic masking can be excluded here as periods of high flow-noise were removed before processing.

Although most of the recorded species are not known to produce sounds, six soniferous species (*P. pollachius, P. flavescens, S. melops, G. paganellus, L. mixtus, Z. punctatus*) were present in the studied assemblages. These sound-producing species were more abundant during periods of high water height and are thus unlikely the sources of the vocal peaks observed at low water height. Small cryptogenic species were also more prevalent during high water height periods. The absence of a corresponding increase in acoustic activity during these periods may also reflect an acoustic detection bias, as sounds produced by small soniferous species may fall below detection thresholds when individuals are distant from the hydrophone. Alternatively, this pattern may also result from a visual detection bias rather than a true change in fish abundance, as these species are typically hidden within the substrate or algal canopy. Under high water conditions, resident and small species, often characterized by limited mobility and high site fidelity, may benefit from expanded vertical space and reduced competition [92], making them more readily detected by divers.

With 26 identified sound types, acoustic diversity exceeded that inferred from the 19 observed species. This discrepancy may indicate the presence of unidentified soniferous species or the production of multiple, yet undocumented sound types by some present species. In fact, comparisons with sounds described in the literature and existing sound databases (e.g. *FishSound* [84], *Macauley Library* [85]) of species present in kelp forests were inconclusive. A non-exhaustive list of macrofaunal species (fish and crustaceans) that could potentially produce the different sound types identified in this study, is presented in Supplementary Table S5.

The contrasting findings between visual and acoustic data indicate that although elevated water levels are associated with a greater physical presence and/or a better detection of individuals, sound production was reduced. Although it remains to be explored, factors like species identity, interspecific interactions, behavioural context, and specific environmental conditions such as strong hydrodynamics are likely to shape sound-producing activities (e.g., foraging, courtship) and their relationship to biological presence.

The high acoustic diversity of biological sounds reported here may reflect the richness of species and biological interactions in kelp forests and aligns with findings from other complex marine habitats recognized as biodiversity hotspots. For example, Desiderà et al. [28] identified 14 different sound types in rocky habitats, Bolgan et al. [29] detected 10 different sound types in a Posidonia seagrass meadow, Di Iorio et al. [34] described 24 different sound types in coralligenous reefs, and Tricas and Boyle [93] associated 85 sound types to 45 fish species in coral reefs. Similar to terrestrial forests, kelp forests exhibit high structural heterogeneity across the canopy, the understory, and the rocky substrate. This complexity promotes the coexistence of different macrofauna assemblages [94], which may in turn foster diverse vocal behaviours and promote acoustic niche partitioning [95].

Acoustic diversity remained stable over the summer period, indicating a consistent acoustic community composition. Diel cycles had nonetheless a strong influence on acoustic relative abundance, richness and community structure, with higher acoustic activity and diversity observed at dusk, dawn and during the night. This is in line with findings reported in other kelp forests and many different marine habitats [28, 57, 96, 97]. Many fish species exhibit daily behavioural cycles [98], including their acoustic behaviour [99, 100]. Elevated nocturnal vocal activity may be associated with reduced predation pressure [101, 102]. Acoustic signalling is widely used by soniferous fish for spawning behaviours, territorial defence or social interactions, behaviours that often peak during nocturnal or crepuscular periods when visual communication is less effective [103–105]. This highlights the value of passive acoustics as a sensitive tool for detecting diel behavioural rhythms in macrofauna and for providing information on nocturnal activities, difficult to assess otherwise.

Beyond tidal and diel effect, other environmental variables showed weak influence on acoustic community composition. This was true both at the community level - affecting overall acoustic relative abundance and richness - and at the level of individual sound types, whose responses to environmental gradients were often inconsistent or marginal. This likely reflects the absence of extreme events, the limited range of environmental variation during the study period and the high natural exposure of kelp forest fauna to rapid fluctuations in water height and hydrodynamics. In such dynamic systems, predictable cyclic drivers may outweigh the influence of factors varying more slowly or within typical seasonal ranges. Temperature for instance, although varying substantially across tidal stages, had only a minor effect on acoustic activity and diversity over the sampling period.

Altogether, these results reveal a strong short-term natural rhythmicity governing acoustic expression, suggesting that fauna in tidal kelp forests are adapted to recurring environmental fluctuations. As this study was limited to the summer period and did not encompass environmental anomalies, the acoustic communities and patterns described here are considered representative of natural variability. However, to develop ecoacoustics as an effective means to follow macofaunal community dynamics under increasing global pressure, identifying sound sources and linking sound types to behaviours remain essential. This is also necessary to understand the discrepancies found between acoustic and taxonomic diversity and to assess to what extent variations in acoustic communities reflect changes in macrofaunal assemblages. The difficulty of assigning calls to species in this study further underlines the importance of expanding global reference sound libraries [106, 107] and integrating passive acoustics with complementarity approaches such as visual observations, synchronized video–acoustic recordings [108] or environmental DNA.

## Conclusion

The patterns of acoustic diversity described here illustrate the potential of ecoacoustics for investigating temporal and environmentally driven dynamics in fish assemblages and behaviours that are often difficult to observe directly. Although variations in acoustic communities and species assemblages did not align, additional integrative studies may allow acoustic communities to serve as effective proxies for monitoring shifts in fish assemblages. This study also underscores that combining these approaches enhances ecological interpretation: visual surveys offer species-level taxonomic resolution during daylight, while passive acoustic monitoring provides continuous, non-invasive insights into the dynamics of fish behaviour and possibly, interactions. The observed short-term variability in acoustic community composition in this tidal kelp forest ecosystem illustrates the natural rhythmicity of its fauna and behaviours and provides an essential baseline against which longer-term ecological changes can be assessed. Establishing this baseline is necessary for future efforts aimed at detecting shifts driven by extreme events such as summer heatwaves and for evaluating how macrofaunal communities respond to increasing global change.

## Electronic supplementary material

Below is the link to the electronic supplementary material.

Supplementary Material 1: Figure S1 to S4 showing the selected days for acoustic analyses, the masking effect on acoustic recordings linked to flow-noise at the hydrophone, the period effect on acoustic abundance and richness and the potential effect of tidal currents.

Supplementary Material 2: Table S1 to S5 providing the 3 datasets used for statistical analyses (acoustic, environmental and UVC), the description of the sound types identified on the study and a list of species that could potentially emit these sound types.

## Data Availability

The data generated and/or analysed during the current study are available from the corresponding author on reasonable request. The dataset supporting the conclusions of this article is included within the additional files.
