# Supplementary material for: Diel and tidal rhythms drive fish acoustic communities in a European kelp forest
Source: BMC Ecol Evol. 2026 May 2;26:47. doi: 10.1186/s12862-026-02521-z (PMC13179613; doi:10.1186/s12862-026-02521-z)
Supplement: Supplementary file 1 — Supplementary Material 1: Figure S1 to S4 showing the selected days for acoustic analyses, the masking effect on acoustic recordings linked to flow-noise at the hydrophone, the period effect on acoustic abundance and richness and the potential effect of tidal currents. [file 12862_2026_2521_MOESM1_ESM.docx]

**Supplementary Information for the manuscript titled:**

**“Diel and tidal rhythms drive fish acoustic communities in a European kelp forest”**

Marine Ethève^1^, Pierre Thiriet^2^, Gaëlle Legras^2^, Philippe Lenfant^1^, François Bourrin^1^, Lucia Di Iorio^1^

^1^ Université de Perpignan Via Domitia, Centre de Formation et de Recherche sur les Environnements Méditerranéens, CNRS, UMR 5110, 52 avenue Paul Alduy, 66860 Perpignan, France.

^2^UAR PatriNat (OFB, MNHN, CNRS, IRD), Station Marine de Dinard - MNHN, 38 rue du Port Blanc – 35 800 Dinard, France.


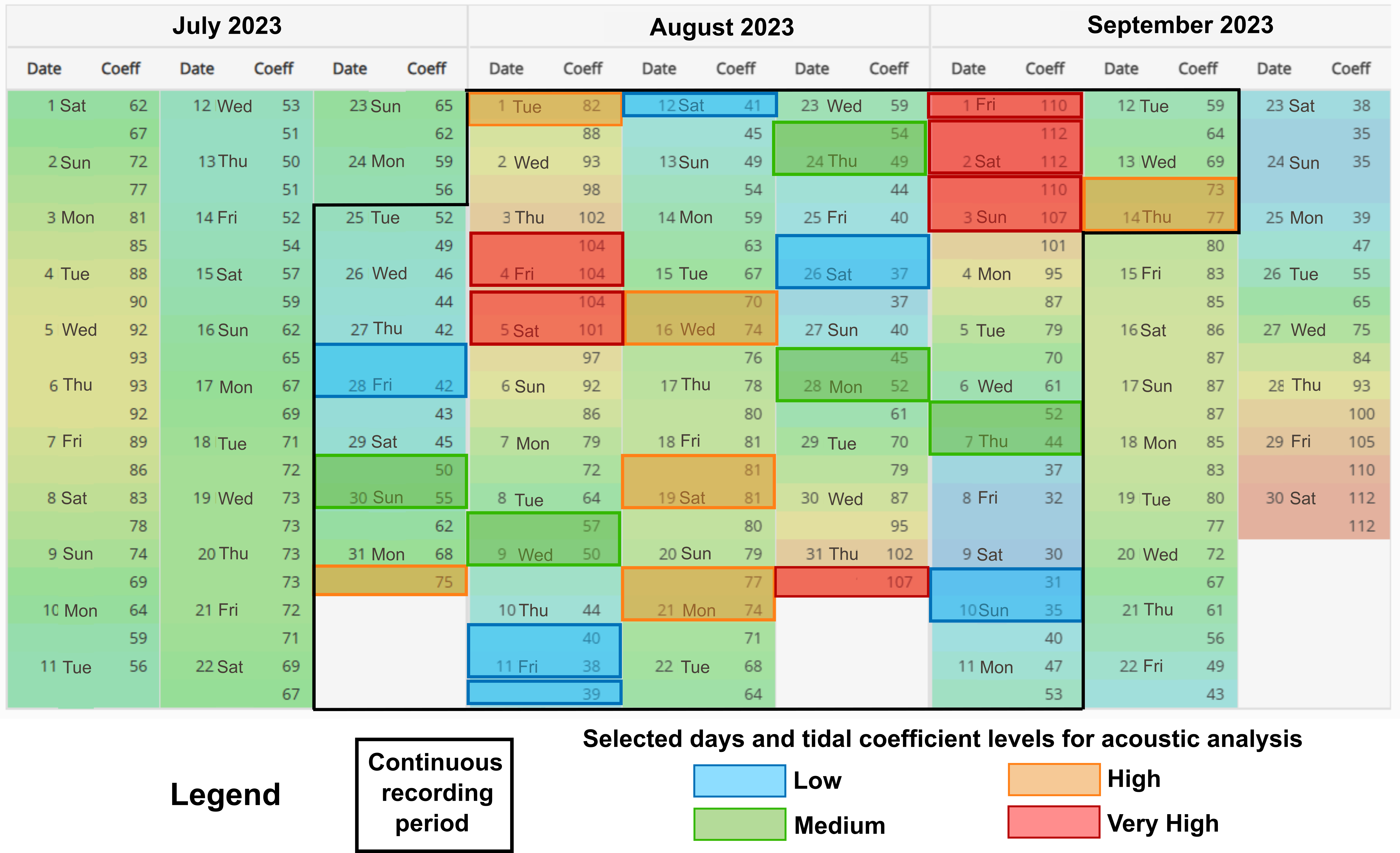
**Fig. S1: Selected days for acoustic analysis, cf. Section 2.2.1 of the manuscript**

**Figure S1.** Overview of selected days and associated tidal coefficient level during summer 2023 for acoustic analysis. Adapted from the SHOM tide calendar website (https://maree.shom.fr/harbor/ROSCOFF/coeff?date=2023-07-28).

**
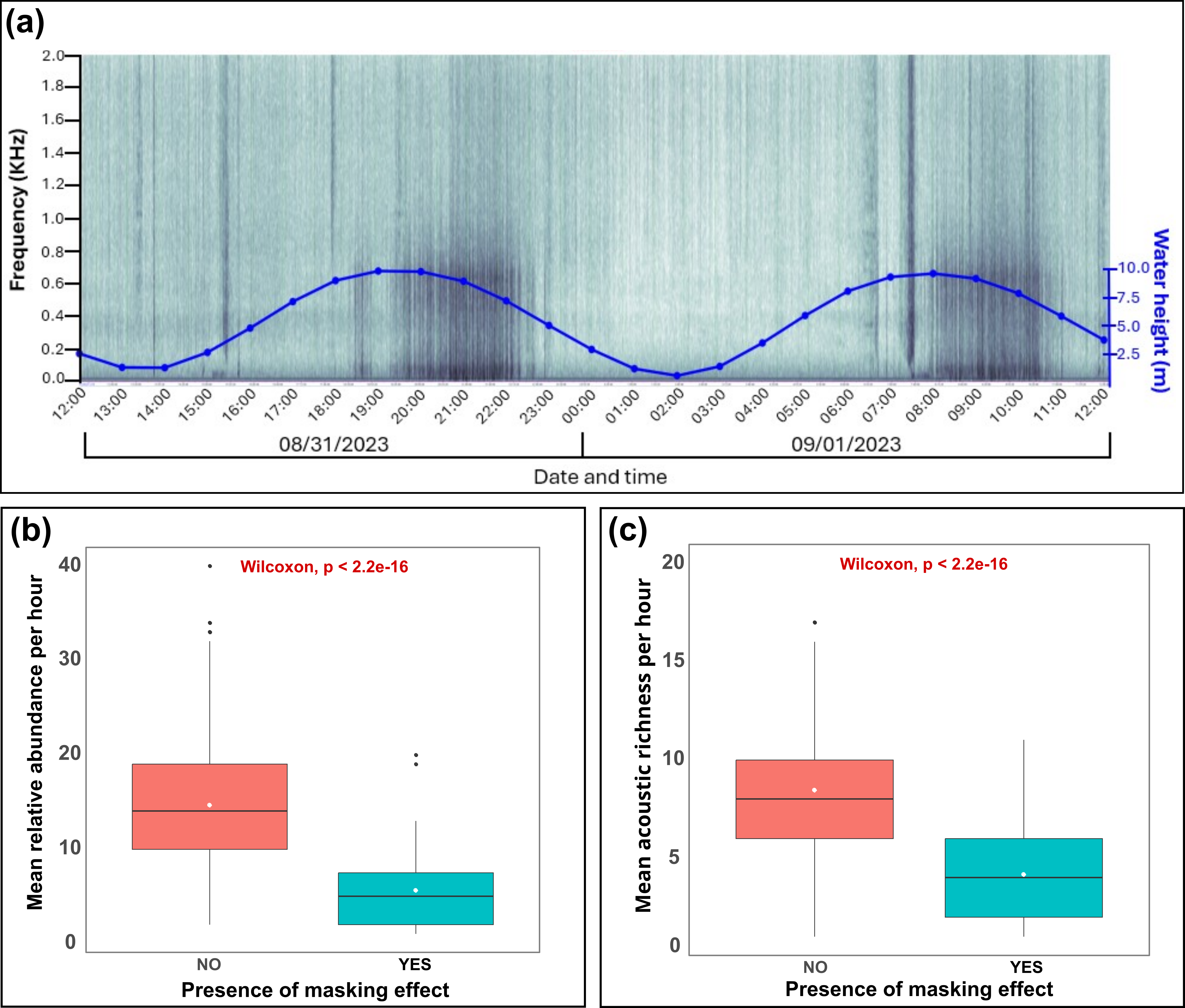
Fig. S2: Illustration of the masking effect linked to flow-noise at the hydrophone, cf. Section 2.2.1 of the manuscript.**

**Figure S2.** Representation of the masking effect during phase of high tide and falling tide for the High and Very high coefficient level. **(a)** Spectrogram from the 08/31/2023 (tidal coefficient = 107) at 12 a.m. to the 09/01/2023 (tidal coefficient = 110) at 12 a.m. with the associated water height (m) in blue. The darkest parts on the spectrogram show the increase in flow-generated noise at the hydrophone during falling tides and the associated hours were not considered in the acoustic dataset for the different analyses. **(b-c)** Boxplot of the mean relative abundance per hour **(b)** and the mean acoustic richness per hour **(c)** to compare the effect of flow-noise on the sound type detection during recording treatment.

**
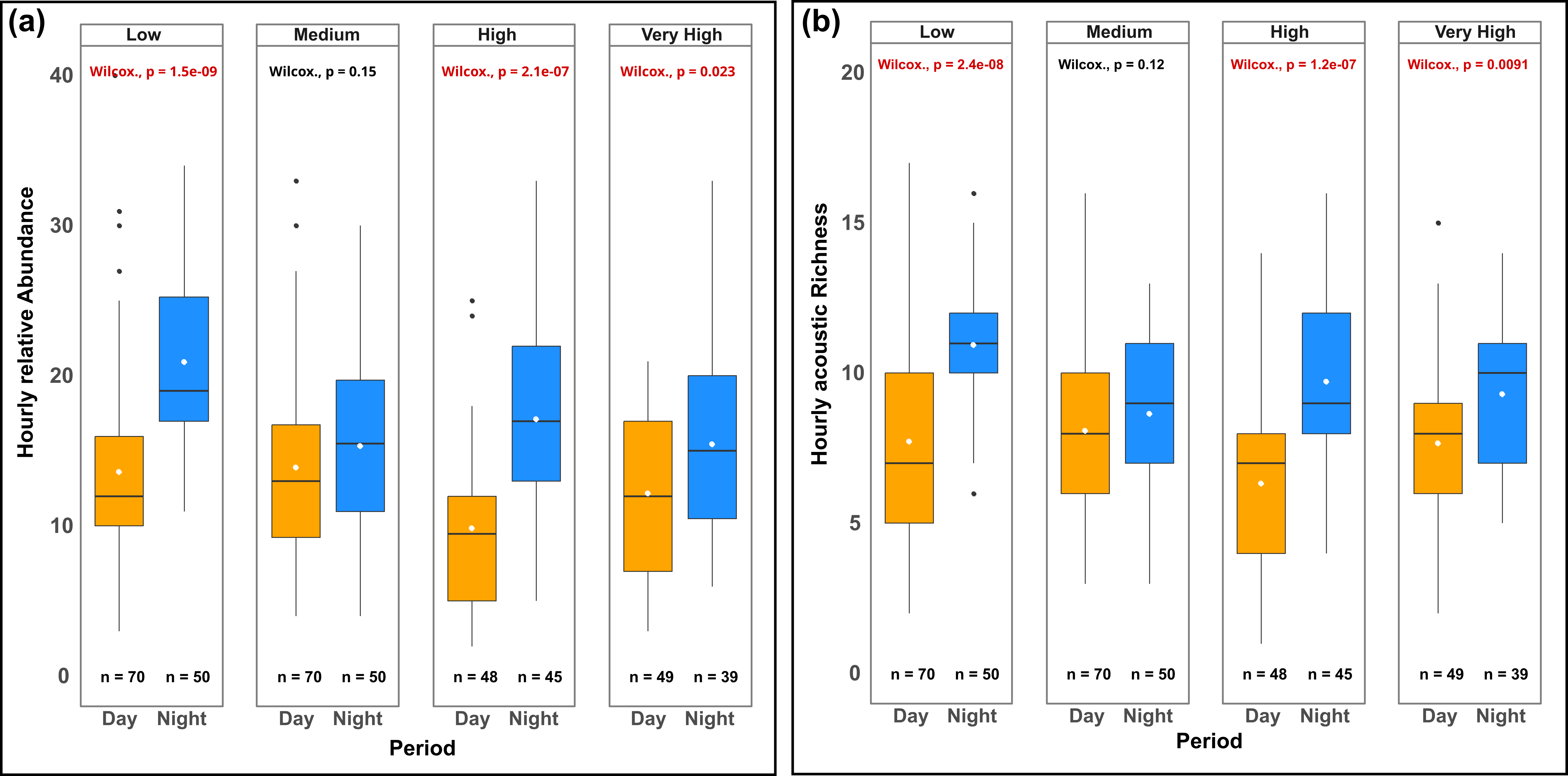
Fig. S3: Visualisation of the period effect, cf. Section 3.3 of the manuscript.**

**Figure S3.** Boxplot visualisation of the Period (Day (orange), Night (blue)) effect on relative abundance and acoustic richness for each Tidal coefficient level (Low, Medium, High, Very high). **(a)** Hourly relative abundance by Period. **(b)** Hourly acoustic richness by Period. White diamonds indicate the mean value by cross-factor. Number of replicates are indicated at the bottom for each crossed factor (n_total_ = 421 hourly replicates). The p-value of the Wilcoxon tests are indicated at the top and the significant means differences are highlighted in red (p-value < 0.05).


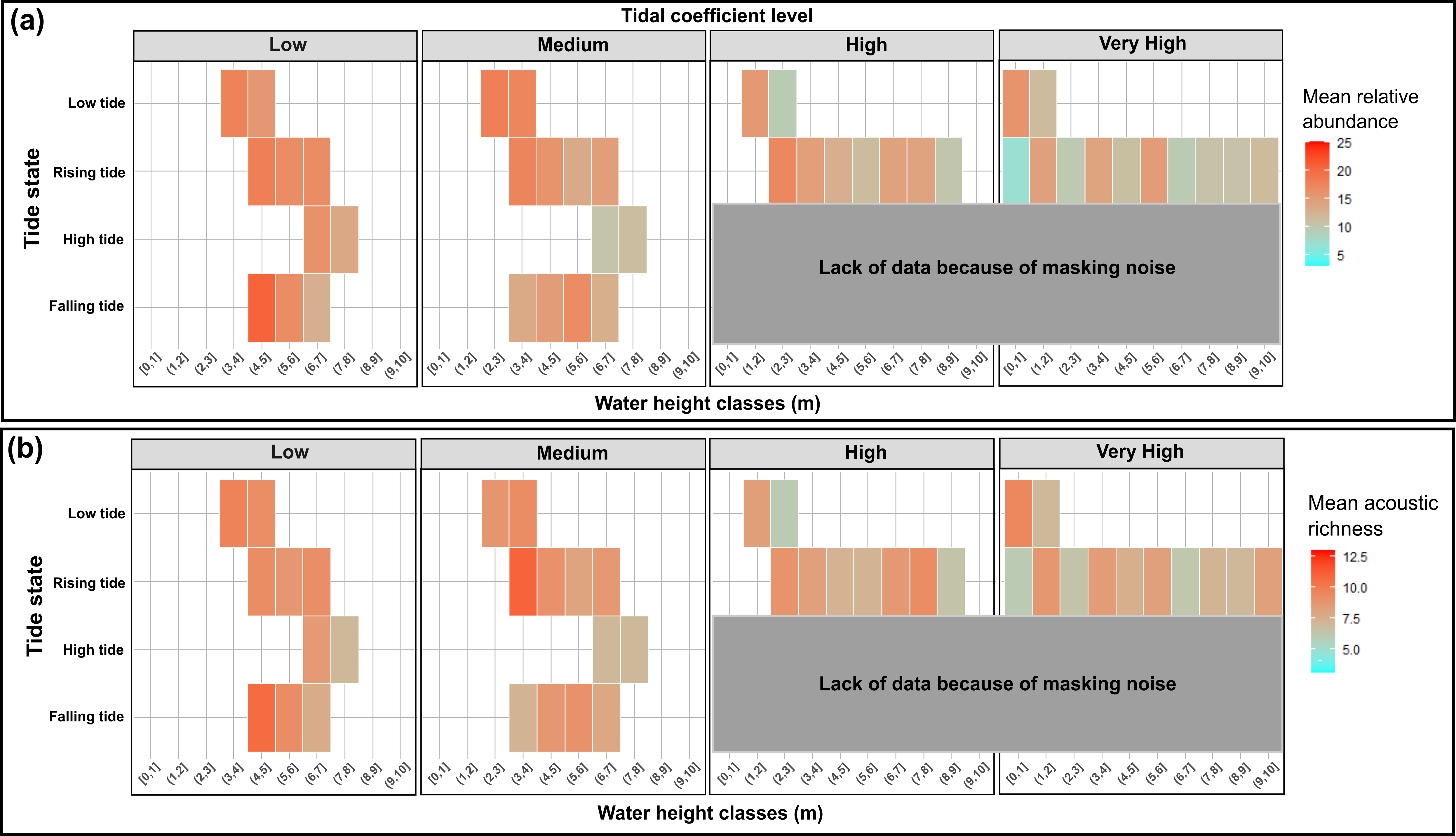
**Fig. S4: Illustration of the potential effect of tidal currents, cf. Section 4 of the manuscript.**

**Figure S4.** Illustration of the potential effect of tidal currents, using tidal phases as proxy. The aim is to compare **(a)** relative abundance and **(b)** acoustic richness for same water height classes and looking for differences between tide state for each tidal coefficient level. For Low and Medium tidal coefficient, periods of tide stall (Low tide and High tide) are longer than for High and Very High tidal coefficient. To define tide states, we used the hour of minimum (Low tide) and maximum (High tide) water height for High and Very High coefficient. We had a window of ± 1 hour around minimum and maximum water height for Low and Medium tidal coefficient to define tide stall. For High and Very High coefficient, the increase of ambient noise during High tide and Falling tide induce a bias in sound detection. The hours with masking noise were not considered (cf. Fig. S2). For the same water height classes, we could observe some important variations of relative abundance and acoustic richness depending on the tide state. For example, in Low tidal coefficient at 4-5 meter of water height, the relative abundance and acoustic richness are higher during falling tide than during Rising or Low tide state. This suggest that the role of tidal currents may be more complex than captured by water height or tidal coefficient alone.
